# Signature of seven cuproptosis-related lncRNAs as a novel biomarker to predict prognosis and therapeutic response in cervical cancer

**DOI:** 10.3389/fgene.2022.989646

**Published:** 2022-09-20

**Authors:** Xinyu Liu, Lei Zhou, Minghui Gao, Shuhong Dong, Yanan Hu, Chunjie Hu

**Affiliations:** ^1^ Department of Obstetrics and Gynecology, The Fourth Affiliated Hospital of Harbin Medical University, Harbin, China; ^2^ NHC Key Laboratory of Molecular Probes and Targeted Diagnosis and Therapy, Harbin Medical University, Harbin, China; ^3^ Department of Orthopedics, The Second Affiliated Hospital of Harbin Medical University, Harbin, China

**Keywords:** cuproptosis, lncRNA, cervical cancer, prognosis, immunotherapy, targeted therapy

## Abstract

**Background:** Given the high incidence and high mortality of cervical cancer (CC) among women in developing countries, identifying reliable biomarkers for the prediction of prognosis and therapeutic response is crucial. We constructed a prognostic signature of cuproptosis-related long non-coding RNAs (lncRNAs) as a reference for individualized clinical treatment.

**Methods:** A total of seven cuproptosis-related lncRNAs closely related to the prognosis of patients with CC were identified and used to construct a prognostic signature via least absolute shrinkage and selection operator regression analysis in the training set. The predictive performance of the signature was evaluated by Kaplan–Meier (K-M) analysis, receiver operating characteristic (ROC) analysis, and univariate and multivariate Cox analyses. Functional enrichment analysis and single-sample gene set enrichment analysis were conducted to explore the potential mechanisms of the prognostic signature, and a lncRNA–microRNA–mRNA network was created to investigate the underlying regulatory relationships between lncRNAs and cuproptosis in CC. The associations between the prognostic signature and response to immunotherapy and targeted therapy were also assessed. Finally, the prognostic value of the signature was validated using the CC tissues with clinical information in my own center.

**Results:** A prognostic signature was developed based on seven cuproptosis-related lncRNAs, including five protective factors (AL441992.1, LINC01305, AL354833.2, CNNM3-DT, and SCAT2) and two risk factors (AL354733.3 and AC009902.2). The ROC curves confirmed the superior predictive performance of the signature compared with conventional clinicopathological characteristics in CC. The ion transport-related molecular function and various immune-related biological processes differed significantly between the two risk groups according to functional enrichment analysis. Furthermore, we discovered that individuals in the high-risk group were more likely to respond to immunotherapy and targeted therapies including trametinib and cetuximab than those in the low-risk group. Finally, CC tissues with clinical data from my own center further verify the robustness of the seven-lncRNA risk signature.

**Conclusion:** We generated a cuproptosis-related lncRNA risk signature that could be used to predict prognosis of CC patients. Moreover, the signature could be used to predict response to immunotherapy and chemotherapy and thus could assist clinicians in making personalized treatment plans for CC patients.

## Introduction

Cervical cancer (CC) is a malignant tumor that affects women’s reproductive systems, posing an exceptional threat to physical and mental health (Arbyn et al., 2020). As reported in the Global Cancer Incidence Report 2020, CC is the fourth most common type of cancer in women, with the fourth highest rate of new cases and fatalities (Sung et al., 2021). In most developing countries, CC is still the top cause of cancer-related death in women (Vaccarella et al., 2017). Although substantial progress has been achieved in the early detection and treatment of CC, there are still many patients with advanced and recurrent disease for whom surgery and radiotherapy or chemotherapy are not suitable, and who have poor prognosis, suggesting that current methods for risk stratification and predicting prognosis based on clinicopathological characteristics of patients may be inadequate (Seol et al., 2014). As a result, new predictive biomarkers for prognosis and drug sensitivity in patients with CC are required.

Cuproptosis is a novel copper-induced form of programmed cell death that was first reported by Tsvetkov et al. (2022). Excessive accumulation of intracellular copper leads to the aggregation of mitochondrial dihydrolipoamide S-acetyltransferase and destabilizes Fe-S cluster proteins, ultimately resulting in copper-induced cell death *via* effects on the tricarboxylic acid cycle. Copper has a crucial role in cancer signaling pathways and antitumor drug resistance (Lelièvre et al., 2020; Ruiz et al., 2021; Shanbhag et al., 2021). The abnormal accumulation of copper in tumor tissues has been the basis for the development of copper-based targeted anticancer agents (Michniewicz et al., 2021; Oliveri, 2022). Copper ion carriers such as disulfiram and elesclomol can function as therapeutic agents in cancer by inducing copper toxicity (Kirshner et al., 2008; Tsvetkov et al., 2019). This represents a new approach to killing tumor cells using the unique action of copper. Moreover, it has been demonstrated that tumor tissues of CC patients have higher copper levels than normal tissues (Margalioth et al., 1983; Zhang et al., 2018). Therefore, factors involved in copper metabolism are potential prognostic markers and therapeutic targets in CC.

Long non-coding RNAs (lncRNAs) are a class of non-coding RNAs whose length exceeds 200 nucleotides. They participate in epigenetic, cellular transcriptional, and post-transcriptional regulation through interactions with proteins, RNA, and DNA (Kapranov et al., 2007; Zimmer-Bensch, 2019). In recent years, many studies have suggested that aberrant lncRNA expression contributes to the progression of malignancy (Prensner and Chinnaiyan, 2011). Moreover, dysregulated lncRNAs are strongly associated with human cancers, including breast, lung, and bile duct cancers and CC(Wang et al., 2019; Li et al., 2020). One study showed that lncRNA XLOC_006390 plays a significant part in cervical carcinogenesis and metastasis owing to its ability to downregulate miR-331-3p and miR-338-3p expression (Luan and Wang, 2018). Furthermore, lncRNAs are involved in regulating the response of cancer cells to antineoplastic drugs and contribute to the emergence of chemoresistance in CC (He et al., 2020).

Although a number of studies have investigated the role of lncRNAs in the occurrence and progression of CC, there has been little systematic analysis of the relationship between lncRNAs and cuproptosis in CC. In this study, we identified lncRNAs associated with cuproptosis and constructed a risk signature, with the goal of predicting survival of CC patients and responsiveness to immunotherapy and targeted treatment.

## Materials and methods

### Data acquisition and processing

Transcriptome RNA (RNA sequencing; RNA-seq) data of 309 samples from CC patients, comprising 306 tumor samples and three paracancerous samples, were obtained from The Cancer Genome Atlas (TCGA; https://portal.gdc.cancer.gov/). For further enriching the normal samples sizes, RNA-seq data of 92 normal samples were downloaded from Genotype-Tissue Expression Project (https://gtexportal.org/home/). The corresponding clinical data were downloaded in BCR XML format. Patients with a follow-up period less than 10 days were excluded, owing to the likelihood of non-cancer mortality, leaving 285 CC patients in the final cohort. Using the Ensembl database (http://asia.ensembl.org/index.html), the Ensembl ID numbers of genes were converted to gene symbols using Perl (http://www.perl.org/). The RNA-seq data were distinguished into mRNA and lncRNA data according to the annotation information in the GENCODE database (https://www.gencodegenes.org/).

### Identification of cuproptosis-related lncRNAs in CC

Searching published studies yielded a total of 19 cuproptosis-related genes, which were included in Supplementary Table S1. Using the R package “limma,” we performed Pearson correlation analysis and identified 460 cuproptosis-related lncRNAs using the criteria |R|>0.3 and *p* < 0.05 (Schober et al., 2018). The correlations between cuproptosis-related lncRNAs and mRNAs were plotted using the R package “ggalluvial.”

### Construction of cuproptosis-related lncRNAs prognostic signature for cervical cancer patients

The entire dataset (*n* = 285) was randomly divided into training (*n* = 143) and test (internal validation, *n* = 142) subsets in a 1:1 ratio. Univariate Cox regression analysis was performed to identify prognosis-related lncRNAs in CC patients (*p* < 0.05). The R package “limma” was used to examine the expression levels of prognosis-related lncRNAs between kidney tumors and paracancerous normal tissues. Then, least absolute shrinkage and selection operator (LASSO) regression analysis was used to shrink the range of gene screening in the training set using R package “glmnet,” and the optimal values of the penalty parameter (*λ*) were determined by ten-fold cross-validation (Friedman et al., 2010). Finally, multivariate Cox regression analysis was performed to establish a prognostic signature. The risk score (RS) was calculated as follows:

$$Risk\;score=\overset{7}{\underset{i=7}{\sum }}\beta_{i}*{\mathrm{Exp}}_{i}$$ (1)

### Estimation and validation of prognostic predictive signature

Samples in the training set were classified into two subgroups (high- and low-risk subgroups) based on their RS, using the median RS as the cut-off. Kaplan–Meier (K-M) survival analysis was used to assess the predictive ability of the cuproptosis-related lncRNA signature using the “survival” and “survminer” R packages. Time-dependent receiver operating characteristic (tROC) curves were generated to estimate the signature’s sensitivity and specificity in predicting 1‐, 3‐, and 5‐year survival of CC patients using the “timeROC” R package. In the validation set, each patient was first assigned an RS based on our signature, and then the set was divided into high and low risk subgroups based on the median RS from the training set. Subsequently, K-M survival and tROC analyses were performed to further confirm the predictive capability and applicability of the prognostic signature. In addition, differences of overall survival associated with clinicopathological characteristics including age, stage, grade, and TNM stage, classified by RS, were compared by survival analysis and log-rank tests.

### Cell cultures and clinical samples collection

Human CC cell lines (HeLa, C-33A and Ca Ski) were obtained from Procell Life Science & Technology Co., Ltd. (Wuhan, China). Human immortalized keratinocytes (HaCaT cells) were obtained from the Chinese Academy of Sciences Cell Resource Center (Shanghai, China). All cells were cultured in Dulbecco’s modified Eagle medium with 10% bovine serum albumin, 0.1 mg/ml streptomycin, and 100 U/ml penicillin (Gibco, Invitrogen, Carlsbad, CA, United States) and incubated in a humidified incubator at 37°C with 5% CO_2_.

A total of 20 pairs of cervical tumor tissues and matched adjacent normal tissues were obtained from patients who underwent radical trachelectomy in the Fourth Affiliated Hospital of Harbin Medical University from 2020 to 2021. All patients were diagnosed with cervical cancer for the first time and did not undergo any antitumor therapy before surgery. The study was authorized by the hospital’s medical ethics committee (2022-ZWLLSC-03) and each patient gave informed consent before the collection. All samples were maintained at −80°C for further study.

### Quantitative real-time PCR

The expression levels of the seven candidate lncRNAs were assessed by qRT-PCR using SYBR Green qPCR. Total RNA was extracted from cervical tumor and normal cells and clinical tissues using the TRIzol RNA extraction reagent (Invitrogen, Waltham, MA, United States). Subsequently, it was reverse-transcribed into cDNA using a reverse transcription kit (Takara). Supplementary Table S2 presents the primer sequences. GAPDH served as an internal control. QuantStudio 3 (Thermo Fisher Scientific, Waltham, MA, United States) was used to perform the qRT-PCR, and the relative expression levels were quantified by the 2−ΔΔCt method.

### External validation

The CC samples with prognostic information in my own center served as external validation. Based on the results of RT-qPCR and the formula of the signature, the RS was calculated and the patients were divided into a high-risk group and a low-risk group according to the median RS. Subsequently, the K-M survival analysis and the univariate and multivariate Cox regression analysis were performed to validate the validity and reliability of the prognostic signature.

### Construction and analysis of the competing endogenous RNA network

First, microRNA (miRNA) files from the miRcode (http://www.mircode.org/) database were used for prediction of lncRNA–miRNA interactions using Perl scripts. Then, three online databases, TargetScan (Thomson et al., 2011), miRDB (Jeggari et al., 2012), and miRTarBase (Huang et al., 2020), were used to predict targets of miRNAs and construct miRNA–mRNA pairs. Finally, a lncRNA–miRNA–mRNA ceRNA network based on the lncRNA–miRNA and miRNA–mRNA pairs was constructed and visualized using Cytoscape 3.9.0 to represent the co-expression network of cuproptosis-related lncRNAs, miRNAs, and mRNAs. The biological functions of target mRNAs in the ceRNA network were explored using Metascape (http://metascape.org/gp/index.html) (Zhou et al., 2019).

### Principal component analysis and functional enrichment analysis

PCA was conducted using the “scatterploted” R package to determine the distributions of patients in different risk groups. Differentially expressed genes (DEGs) between the two risk groups were analyzed using the “limma” R package with criteria of |log2 fold change (FC)|>1 and *p* < 0.05. Kyoto Encyclopedia of Genes and Genomes (KEGG) and gene ontology (GO) analyses (including biological processes, cellular components, and molecular functions) were performed with the “clusterProfiler” R package to explore the underlying pathways and functions of DEGs.

### Single-sample gene set enrichment analysis and immunotherapy

The activities of 13 immune-related pathways and the infiltration scores of 16 immune cell types in CC were analyzed using the ssGSEA algorithm in the “gsva” R package. In addition, survival analysis was performed to explore the survival significance of different immune checkpoints in patients with high- and low-risk scores.

### Correlation analysis for targeted therapy

Analysis of the half-maximal inhibitory concentration (IC50) values of typical chemotherapeutic agents in the TCGA database was conducted using the “pRRophetic” R package (Geeleher et al., 2014). We used the Wilcoxon signed-rank test to detect the relative differences in IC50 between the low-risk group and the high-risk group.

### Statistical analysis

All data analysis was performed using the Perl data language (http://www.perl.org/), R software (version 4.1.1, https://www.r-project.org/) and GraphPad version 8. Wilcoxon rank-sum test were used to compare the difference between two groups, and the difference between two or several groups was compared with the Kruskal-Wallis test and statistical significance was defined as *p* < 0.05.

## Results

**FIGURE 1:**
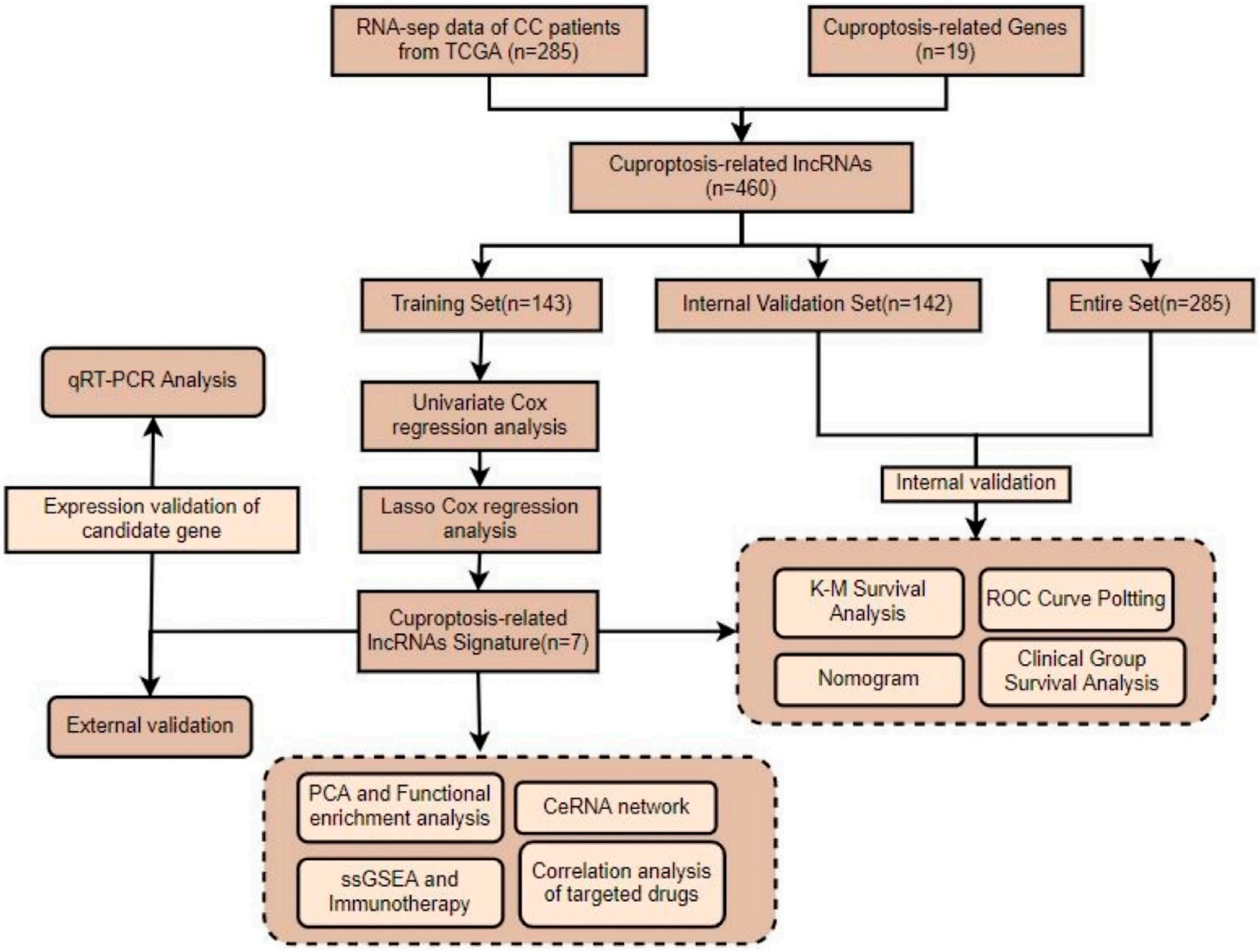
Flow chart showing the scheme of our study on cuproptosis-related lncRNAs in CC.

A flow chart of the overall study is presented in Figure 1. The entire cohort of patients (*n* = 285) was randomly assigned according to a 1:1 ratio into training and internal validation groups (Table 1). Validation of the results from the training set was performed using the internal validation set as well as the entire set. The CC samples (*n* = 20) with prognostic information in my own center served as external validation.

**TABLE 1:** Characteristics of training set, internal validation set, and entire set.

Characteristics	Training set	Internal validation set	Entire set	*p* value
	N = 143	N = 142	N = 285	
	Number (%)	Number (%)	Number (%)	
Age				0.2576
≤65	130 (90.9)	122 (85.9)	252 (88.4)	
>65	13 (0.1)	20 (14.1)	33 (11.6)	
Grade				0.8908
1–2	76 (53.1)	69 (48.6)	145 (50.9)	
3	58 (40.6)	54 (38.0)	112 (39.3)	
Unknown	9 (6.3)	19 (13.4)	28 (9.8)	
Stage				0.2610
I-II	49 (33.3)	48 (33.8)	97 (34.0)	
III-IV	1 (0.7)	3 (2.1)	4 (1.4)	
Unknown	93 (65.0)	91 (64.1)	184 (64.6)	
Tumor size				0.6561
T1+T2	104 (72.7)	96 (67.6)	200 (70.2)	
T3+T4	11 (7.7)	15 (10.6)	26 (9.1)	
Unknown	28 (19.6)	31 (21.8)	59 (20.7)	
Lymph node metastasis				0.6981
N0	65 (45.4)	60 (42.3)	125 (43.9)	
N1	26 (18.2)	28 (19.7)	54 (18.9)	
Unknown	52 (36.4)	54 (38.0)	106 (37.2)	
Distant metastasis				0.7564
M0	55 (38.5)	51 (35.9)	106 (37.2)	
M1	4 (2.8)	6 (4.2)	10 (3.5)	
Unknown	84 (58.7)	85 (59.9)	169 (59.3)	
Death				1
No	107 (74.8)	107 (75.4)	214 (75.1)	
Yes	36 (25.2)	35 (24.6)	71 (24.9)	

Statistical analysis in age, grade, stage, tumor size, lymph node metastasis, distant metastasis, and Death between training set and internal validation set.

### Construction of cuproptosis-related lncRNA prognostic signature

**FIGURE 2:**
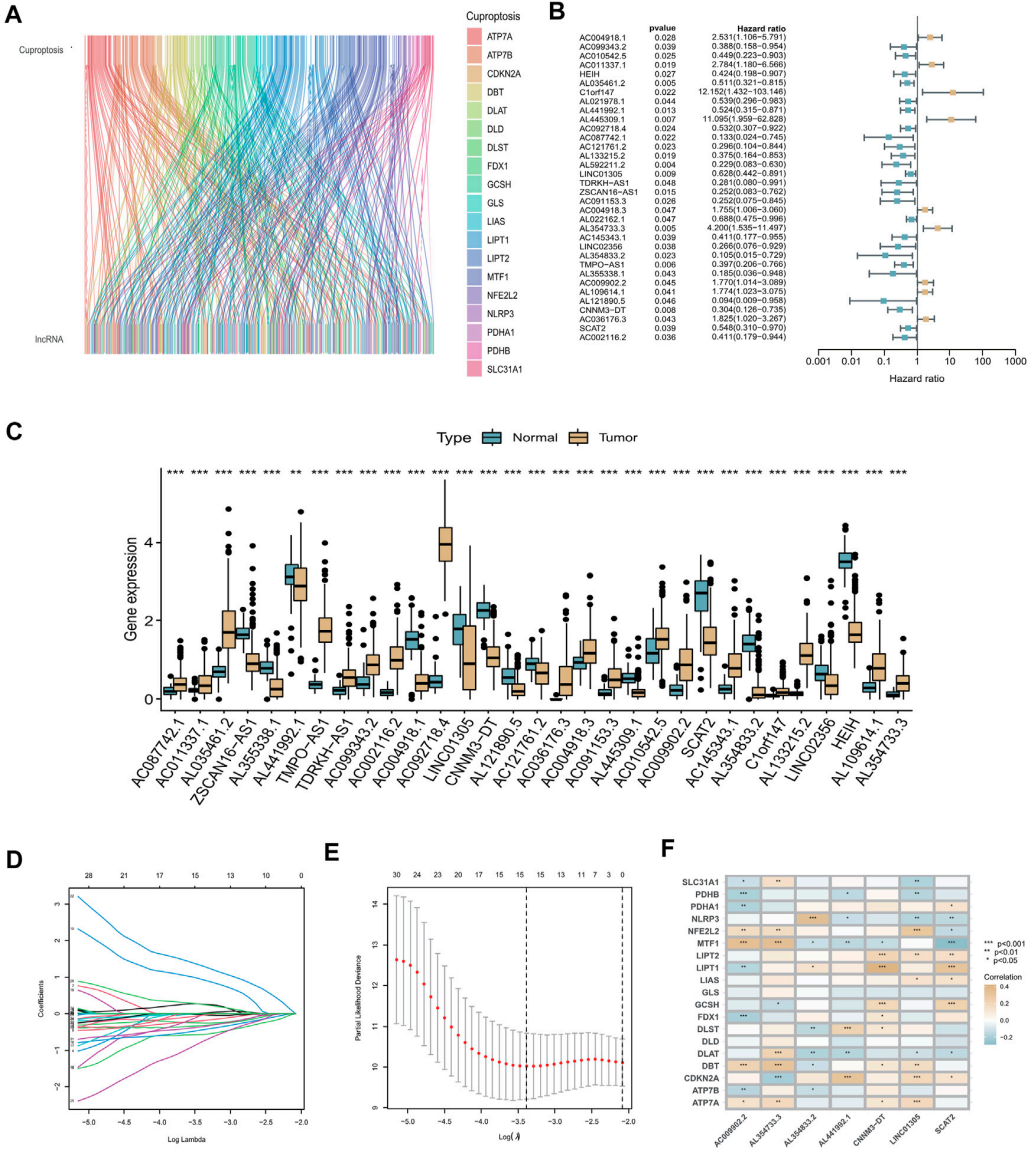
Construction of cuproptosis-related lncRNA prognostic signature. **(A)** Sankey diagram used to visualize the co-expression network of cuproptosis-related lncRNAs. **(B)** Forest plot representing the results of the univariate Cox regression analysis. **(C)** Expression levels of 34 cuproptosis-related lncRNAs that differed between tumor tissue and normal tissue. Normal tissue is shown in blue, tumor tissue in yellow. Adjusted *p*-values are indicated (*p < 0.05, **p < 0.01, ***p < 0.001). **(D)** LASSO coefficient profiles of the 34 cuproptosis-related lncRNAs. **(E)** Options for the parameter (*λ*) in ten-fold cross-validation. **(F)** Correlation analysis between the seven cuproptosis-related lncRNAs and 19 cuproptosis-related genes.

By analyzing RNA-seq data of CC patient tissue samples from TCGA in conjunction with the GENCODE website, we identified 460 lncRNAs associated with cuproptosis among the 16,876 lncRNAs annotated in TCGA and 19 cuproptosis-related genes (|Pearson R |>0.3 and *p* < 0.05) using Pearson correlation analysis. A co-expression network of the 460 cuproptosis-related lncRNA and mRNAs was constructed and displayed using a Sankey diagram (Figure 2A). By univariate Cox analysis performed in the training set, we identified 34 lncRNAs associated with CC prognosis, including 25 low-risk lncRNAs (hazard ratio (HR) < 1) and nine high-risk lncRNAs (HR > 1) (Figure 2B). To determine whether these 34 cuproptosis-related lncRNAs were involved in CC development, we compared their expression levels between normal and tumor tissue samples. According to the boxplot, all these cuproptosis-related lncRNAs showed significantly differential expression between the two groups. (*p* < 0.05, Figure 2C). LASSO–Cox regression analysis was performed to simplify the signature; 15 cuproptosis-related lncRNAs were identified using the minimum partial likelihood deviance. (Figures 2D,E). Subsequently, seven lncRNAs significantly associated with prognosis were identified by multivariable Cox regression analysis, namely AL441992.1, LINC01305, AL354733.3, AL354833.2 AC009902.2, CNNM3-DT, and SCAT2 (Supplementary Table S3). Of these prognostic factors, AC009902.2 and AL354733.3 were identified as risk factors (HR > 1), whereas AL441992.1, LINC01305, AL354833.2, CNNM3-DT, and SCAT2 were protective factors (HR < 1). A correlation heatmap was constructed to represent the correlations between the seven candidate lncRNAs and cuproptosis-related genes (Figure 2F). A risk score (RS) for patients with CC was calculated based on the expression levels of the seven candidate lncRNAs and their Cox coefficients as follows:

RS = (−0.7587 * ${\mathrm{Exp}}_{AL441992.1}$ ) + (−0.8764 * ${\mathrm{Exp}}_{LINC01305}$ ) + (2.5069 * ${\mathrm{Exp}}_{\;AL354733.3}$ ) + (−2.9154 * ${\mathrm{Exp}}_{AL354833.2}$ ) + (0.6460 * ${\mathrm{Exp}}_{AC009902.2}$ ) + (−0.8982 * ${\mathrm{Exp}}_{CNNM3-DT}$ ) + (−0.6404 * ${\mathrm{Exp}}_{SCAT2}$ ).

### The cuproptosis-related seven-lncRNA signature performed well in predicting prognosis of cervical cancer patients

**FIGURE 3:**
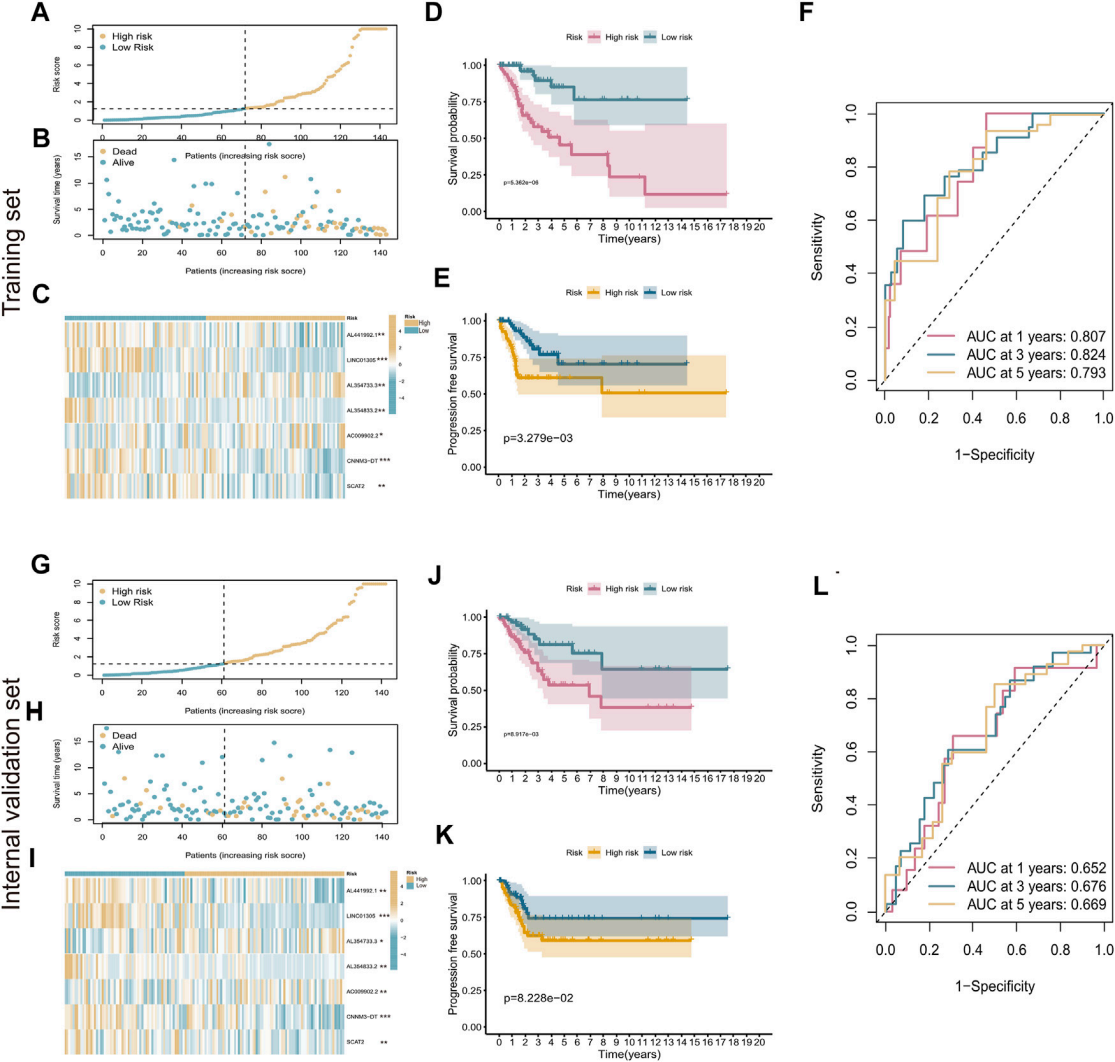
Evaluation of cuproptosis-related seven-lncRNA prognostic signature. **(A)** Risk curves based on the risk scores in high-risk and low-risk groups. **(B)** Scatter plot showing each patient’s survival status. Blue represents survival, yellow represents death. **(C)** Heatmap showing the expression levels of cuproptosis-related lncRNAs in high-risk and low-risk groups. **(D,E)** Kaplan–Meier survival analysis showing the difference in patient prognosis between the high-risk and low-risk groups with respect to OS **(D)** and PFS **(E)**. **(F)** Time-dependent ROC curves for 1‐, 3‐, and 5‐year OS. **(G–L)** Evaluation of the prognostic ability of the signature in the internal validation set. *p < 0.5; **p < 0.01; ***p < 0.001.

According to the median RS value, CC patients were categorized into high-risk and low-risk groups. Risk curves and scatter plots were used to illustrate the survival status of patients with CC. As shown in Figures 3A,B, the number of deaths increased as the risk score increased. The expression heatmap of the seven cuproptosis-related lncRNAs showed that AC009902.2 and AL354733.3 were strongly overexpressed in the high-risk group, whereas AL354833.2, AL441992.1, CNNM3-DT, LINC01305, and SCAT2, as protective factors, were overexpressed in the low-risk group (*p* < 0.05, Figure 3C).

K-M survival analysis showed that patients with high-risk scores had shorter survival times and shorter progression-free survival (PFS) than those with low-risk scores (*p* < 0.05, Figures 3D,E). ROC curves were used to evaluate the predictive value of the risk signature. The AUC value under the ROC curve reached 0.807 at the first year, 0.824 at the third year, and 0.793 at the fifth year (Figure 3F). The prognostic ability of the signature was also evaluated using the internal validation set and the entire set separately. The K-M results were the same as those obtained in the training set (Figures 3G–K). The AUC values for 1-year, 3-year, and 5-year survival were 0.652, 0.676, and 0.669 for the internal validation set (Figure 3L) and 0.724, 0.757, and 0.741 for the entire set, respectively (Supplementary Figure S1). These results demonstrate that the prognostic signature of cuproptosis-related lncRNAs performed well in predicting the prognosis of patients with CC.

### The risk score was strongly correlated with clinicopathological factors of cervical cancer

**FIGURE 4:**
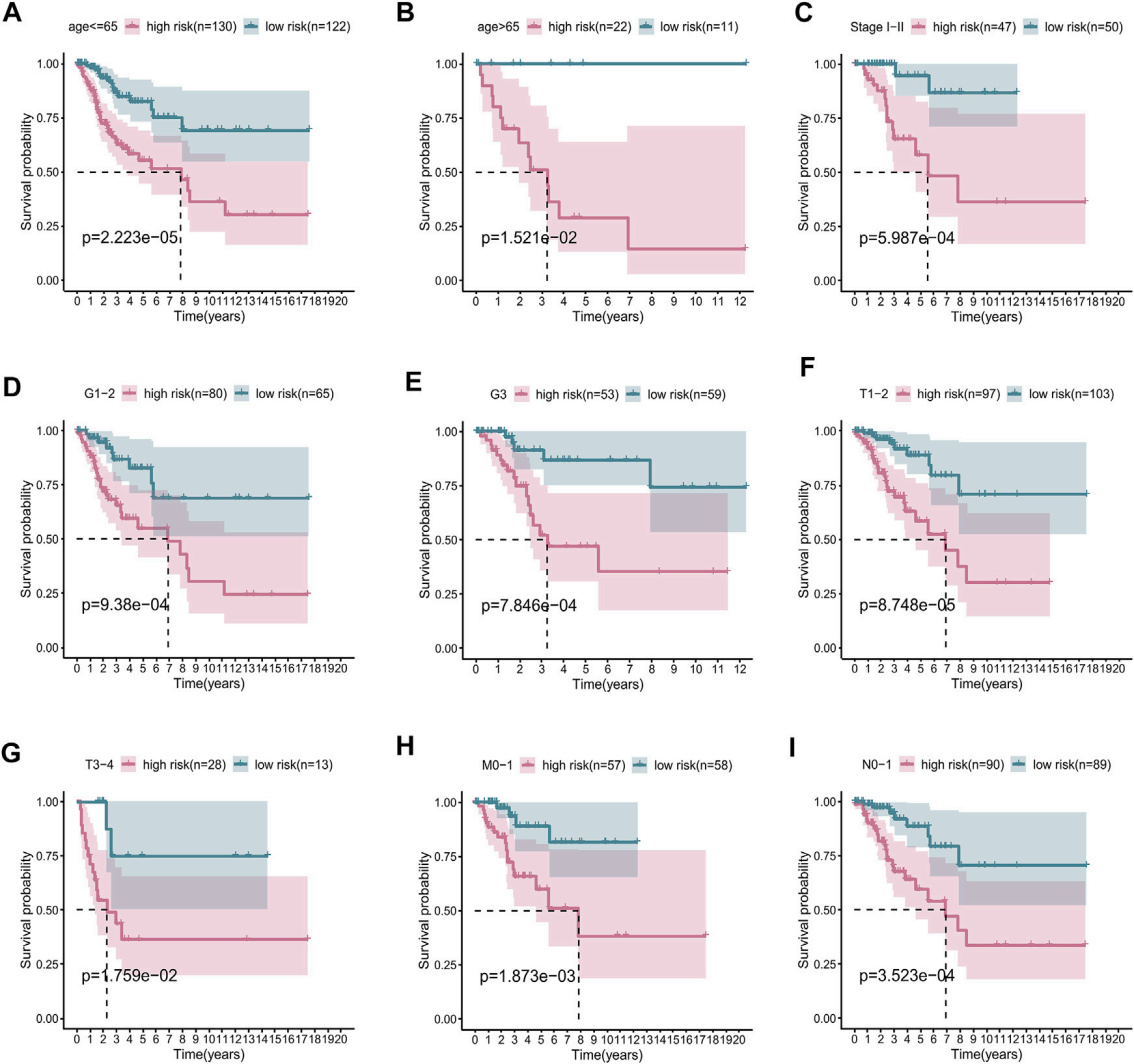
Subgroup survival analysis adjusted by age, grade, stage, T, N, and M. **(A)** age ≤65 years; **(B)** age >65 years; **(C)** stages I–II; **(D)** grades 1–2; **(E)** grade 3; **(F)** T1–2; **(G)** T3–4; **(H)** M0–1; **(I)** N0–1.

To determine whether the risk score could accurately predict the overall survival (OS) of patients, we performed a stratification analysis on a variety of clinicopathological parameters, including age, AJCC stage, grade, and pathological TMN stage. As shown by the survival curves in Figure 4, compared with patients with low-risk scores, those with high-risk scores had worse prognosis in the younger (≤65 years old, *p* < 0.001), older (>65 years old, *p* = 0.008), stage I–II (*p* < 0.001), lower grade (grade 1–2, *p* < 0.001), higher grade (grade 3, *p* < 0.001), T1–2 (*p* < 0.0015), and T3–4 (*p* = 0.006) subgroups. These results further confirm that our seven-lncRNA signature is a reliable tool for predicting the survival of CC patients. Besides, we also investigated the association between RS and various clinicopathological variables, such as age, grade, AJCC-stage and T stage. The results exhibited in Supplementary Figure S2 showed that the RS was significantly positively correlated with these clinical factors.

### The predictive nomogram based on risk score possessed excellent prediction performance

**FIGURE 5:**
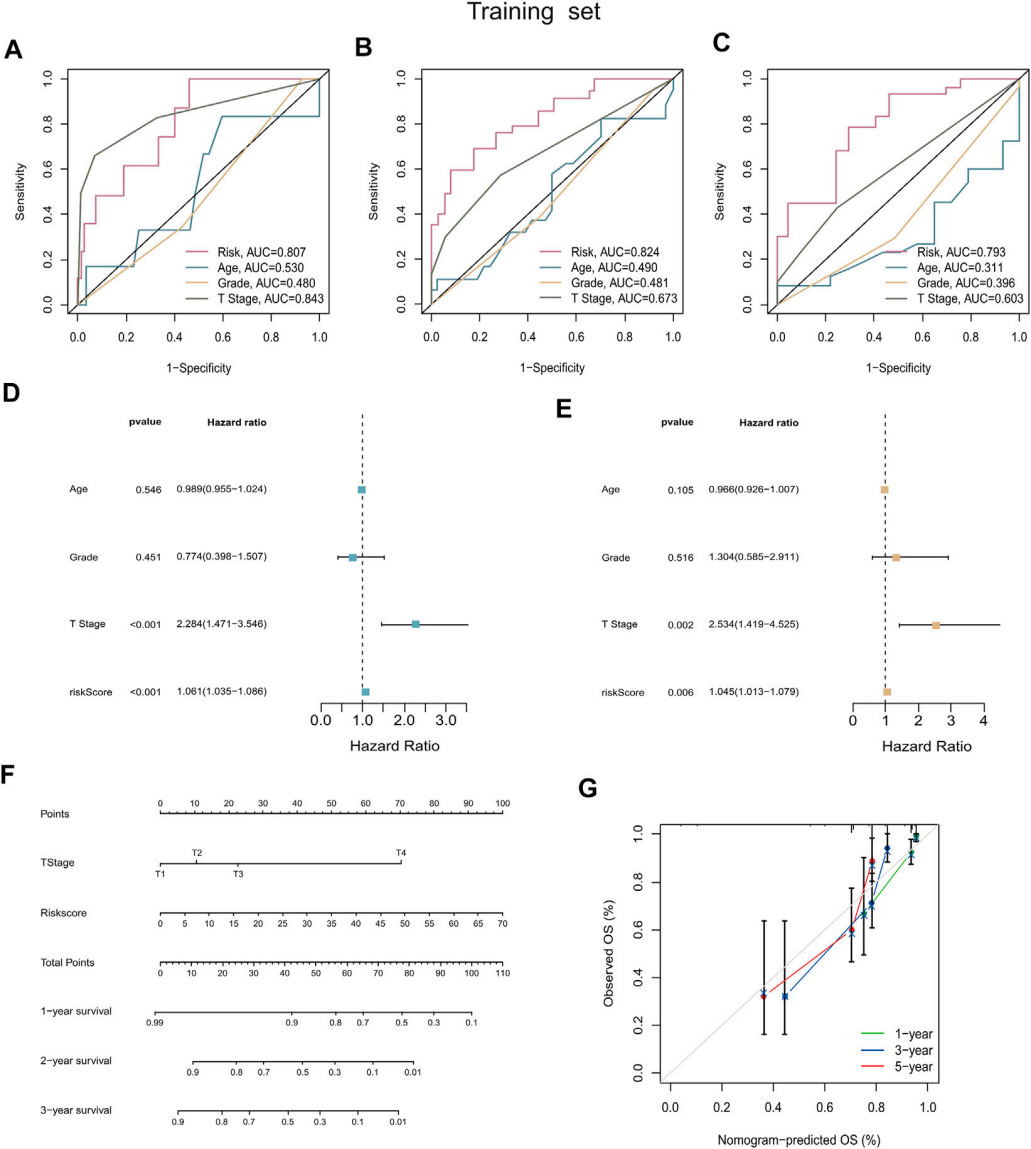
Construction and validation of the prognostic nomogram. **(A–C)** AUC values of nomogram points at 1-year, 3-years, and 5-years were compared with other clinical parameters, including age, grade, stage, and T stage. **(D,E)** Forest plots summarizing the results of univariate and multivariate Cox analyses of risk scores and clinicopathological features. **(F)** The nomogram was plotted based on the signature and T Stage. **(G)** Calibration plot for internal validation of the nomogram. Survival is depicted on the *Y*-axis, and nomogram-predicted survival is shown on the *X*-axis. Besides the dashed diagonal line, green, blue, and red lines represent the 1-year, 3-year, and 5‐year observed nomograms.

As was shown in Figures 5A–C, the ROC curves demonstrated that the risk score possessed better prediction performance than the other clinical parameters in all sets. Subsequently, we performed the univariate and multivariate Cox regression analyses on the training set and the internal validation set (Supplementary Figure S3), as well as the entire set (Supplementary Figure S4). The resulting forest plots demonstrated that the prognostic signature could independently predict the outcome of CC patients. Univariate Cox analysis in the training set showed that T stage (*p* < 0.01) and risk score (*p* < 0.01) were highly correlated with survival in CC, with an HR of 1.061 for the risk score (95% CI: 1.035–1.086) (Figure 5D). Furthermore, multivariate Cox regression confirmed that the risk score (HR: 1.045, 95% CI: 1.013–1.079) was significantly related to CC survival (*p* = 0.006), validating the ability of our prognostic signature to serve as an independent predictive factor (Figure 5E). Next, we used the risk score and other clinicopathological parameters to produce a compound nomogram for predicting the OS of CC patients at 1, 3, and 5 years (Figure 5F). The calibration curves for 1-, 3-, and 5-year OS indicated that the predictions of the prognostic signature were reliable and accurate (Figure 5G).

### Principal component analysis and functional enrichment analysis

**FIGURE 6:**
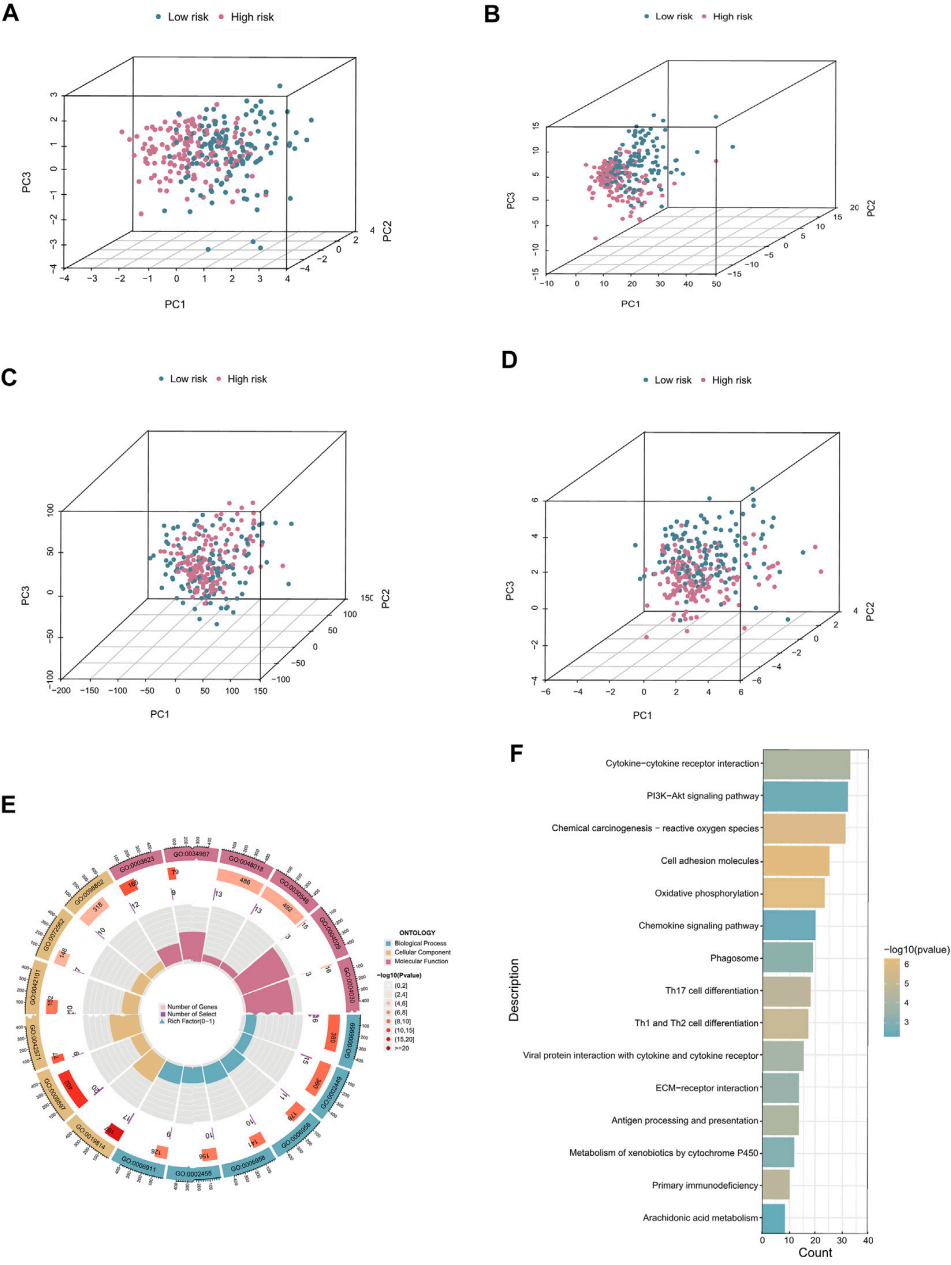
PCA and functional enrichment analysis. **(A–D)** PCA plots based on risk score, cuproptosis-related lncRNAs, cuproptosis-related coding genes, and all risk genes. **(E)** GO biological process, cellular component, and molecular function enrichment. **(F)** KEGG pathway analysis results.

PCA was conducted to investigate the distribution of patients based on different patterns. We found that the prognostic cuproptosis-related seven-lncRNA signature could distinguish low- and high-risk patients more clearly compared with all cuproptosis-related lncRNAs, cuproptosis-related mRNAs, or the all-genes set (Figures 6A–D). Subsequently, to explore the biological functions and pathways of DEGs (Supplementary Table S5) between the two risk groups, we performed GO and KEGG enrichment analyses. The top 18 results, with detailed genetic and pathway information, are listed in Supplementary Table S4.

In the GO analysis, the main biological processes in which the cuproptosis-related lncRNAs were enriched were humoral immune response, leukocyte-mediated immunity, and lymphocyte-mediated immunity. Molecular function enrichment was mainly in receptor-ligand activity, signaling receptor activator, and activity antigen binding, and the main cellular components were immunoglobulin complex, the outer side of the plasma membrane, and plasma membrane signaling receptor complex (Figure 6E). In addition, KEGG analysis showed that cuproptosis-related lncRNAs were enriched in immune molecular pathways including cytokine–cytokine receptor interaction, cell adhesion molecules, and oxidative phosphorylation (Figure 6F).

### Construction of a cuproptosis-related ceRNA regulatory network in cervical cancer

**FIGURE 7:**
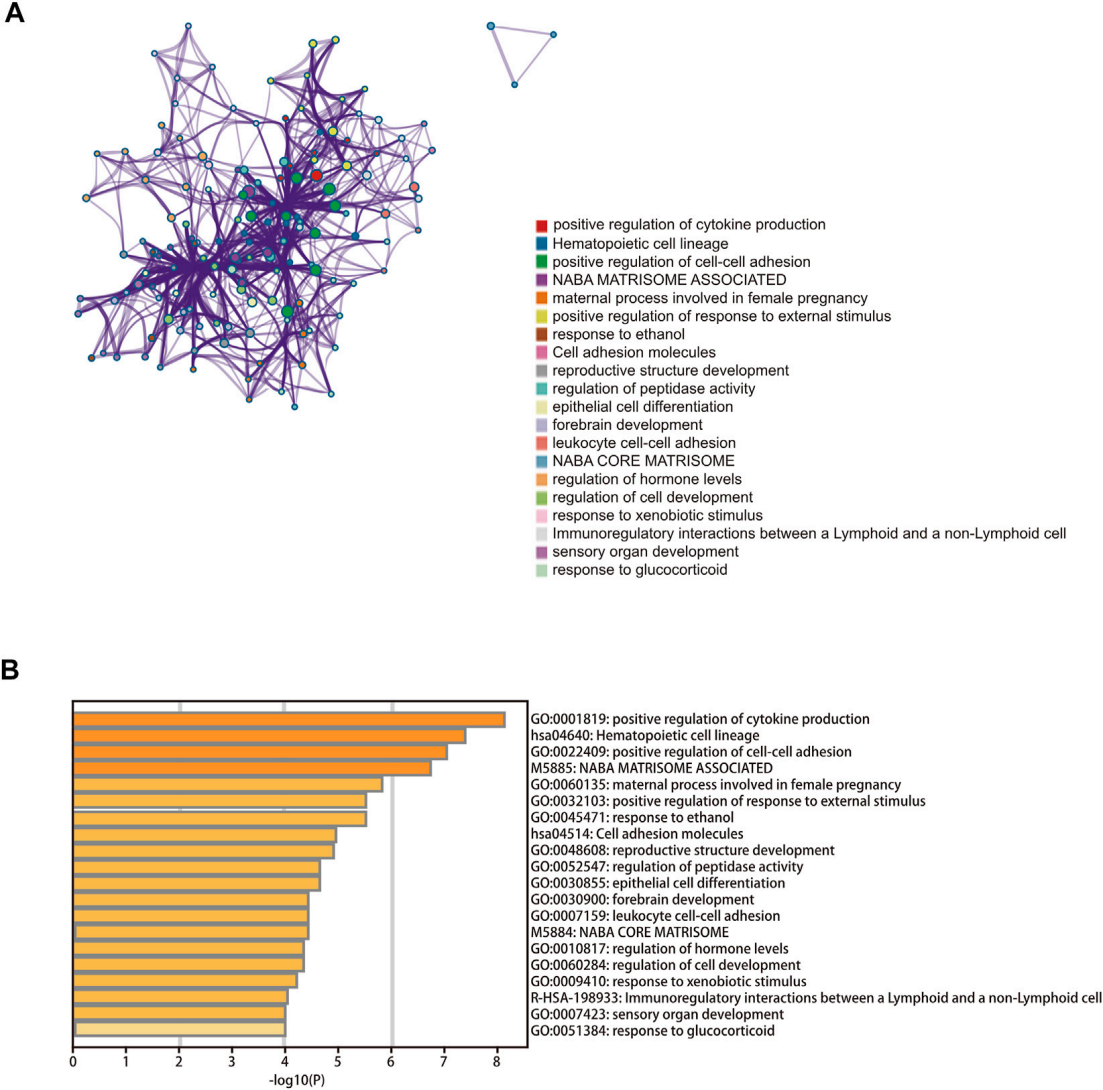
Functional enrichment analysis. **(A)** Bar graph of enriched terms across the 100 target mRNAs. **(B)** Network of enriched terms colored according to cluster ID.

We constructed a lncRNA–miRNA–mRNA regulatory network to explore how the cuproptosis-related lncRNAs regulate the expression of genes involved in cuproptosis by functioning as miRNA sponges in CC. A total of 358 lncRNAs associated with cuproptosis were screened as differently expressed between normal and tumor tissue. Subsequently, 20 lncRNAs and 43 target miRNAs were extracted from the online miRcode database. Next, we extracted 100 targeting mRNAs by integrating the results from three online tools as well as the DEGs between the two risk groups. Finally, the lncRNA–miRNA–mRNA co-expression network was visualized (Supplementary Figure S5; Supplementary Table S6). In order to further explore the biological functions of the target mRNAs in the ceRNA network, a functional enrichment analysis was conducted using the online tool Metascape. According to the results, mRNAs in the ceRNA network were involved in positive regulation of cytokine production, positive regulation of cell–cell adhesion, immunoregulatory interactions between a lymphoid and a non-lymphoid cell, and leukocyte cell–cell adhesion (Figures 7A,B; Supplementary Table S7).

### ssGSEA and immunotherapy

**FIGURE 8:**
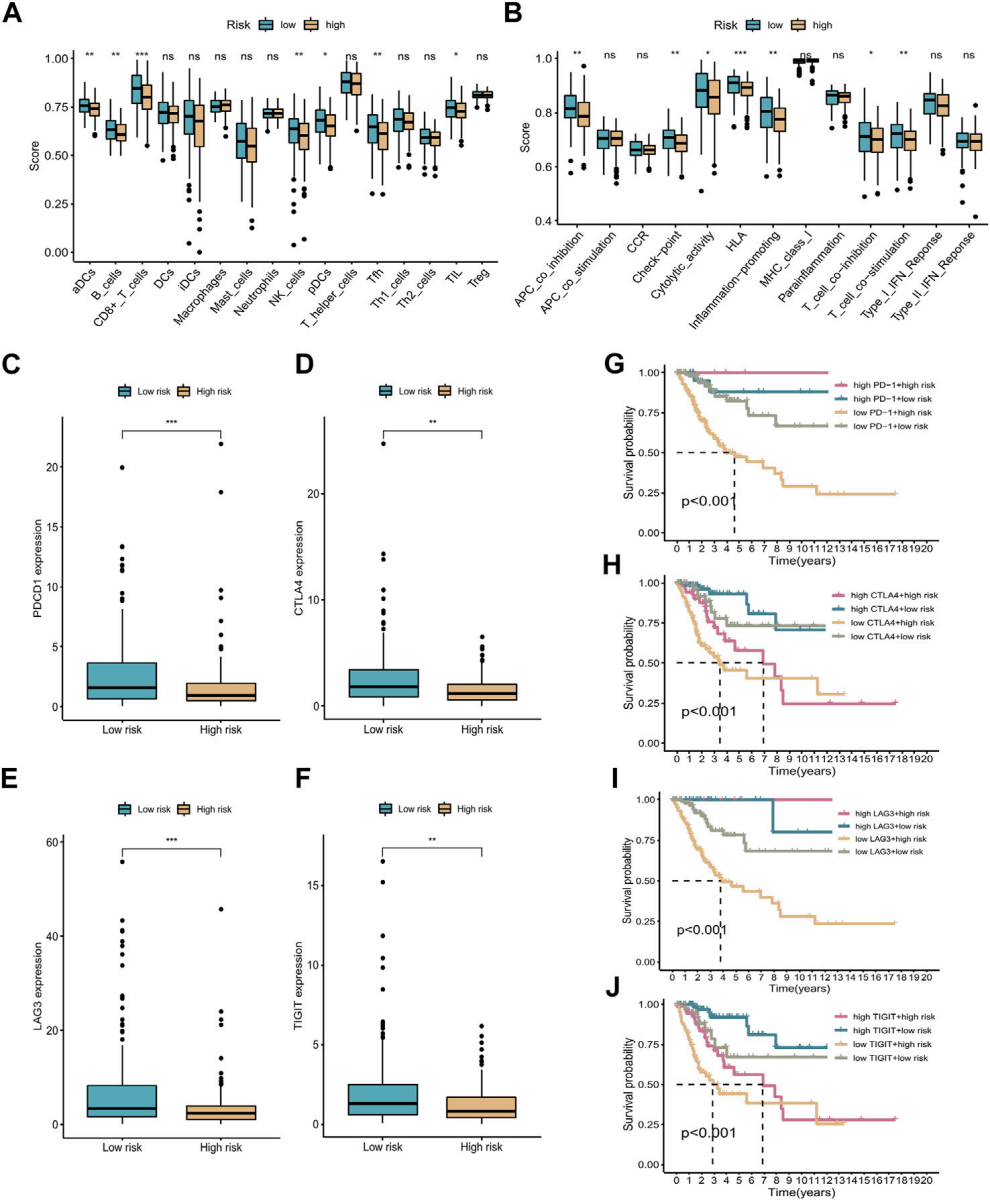
ssGSEA and immunotherapy. **(A,B)** Boxplots of relative levels of infiltration of 16 immune cells **(A)** and 13 immune-related pathways **(B)** in low- and high-risk groups. Adjusted *p*-values (ns, not significant; *p < 0.05, **p < 0.01, ***p < 0.001). **(C–F)** Four immune checkpoints were differentially expressed between the high- and low-risk groups: PD-1, CTLA-4, LAG-3, and TIGIT. **(G–J)** K-M curves comparing OS rates for four patient groups stratified according to the cuproptosis-related lncRNA signature and PD-1, CTLA-4, LAG-3, and TIGIT expression.

For further analysis of the correlation between different groups and immune status, we used ssGSEA to quantify enrichment scores for different immune cell subsets and related functions or pathways. In the low-risk group, several immune cell types were significantly different from those in the high-risk group (e.g., aDCs, B cells, CD8^+^ T cells, NK cells, pDCs, Tfh cells, and TILs) (*p* < 0.05, Figure 8A). In addition, immune functions, including APC co-inhibition, checkpoints, HLA, T cell co-inhibition, and T cell co-stimulation, were more active in the low-risk group (*p* < 0.05, Figure 8B). The above results illustrate that the seven cuproptosis-related lncRNAs are closely related to immune status in CC. Immunotherapy represents a new approach to the treatment of cancer. There is significant evidence that targeting the programmed death 1 (PD-1) and cytotoxic T lymphocyte-associated protein 4 (CTLA-4) immune checkpoints is an effective method of treating CC, and several other checkpoint inhibitors are currently being developed. Four common immune checkpoint molecules, PD-1, CTLA-4, LAG-3, and TIGIT, were selected to explore the relationship between the risk signature and response to immunotherapy. The corresponding immune checkpoint genes were highly expressed in the low-risk group of patients, indicating that immunotherapy may be more suitable for patients in the low-risk group (see boxplots in Figures 8C–F). We also divided the patients into four groups based on their risk scores and expression levels of immune checkpoint genes. The K-M survival curves showed that patients with low-risk scores and high immune gene expression had better prognosis compared with patients with high-risk scores and low immune gene expression (Figures 8G–J).

### The signature could predict the therapeutic response of cervical cancer patients

**FIGURE 9:**
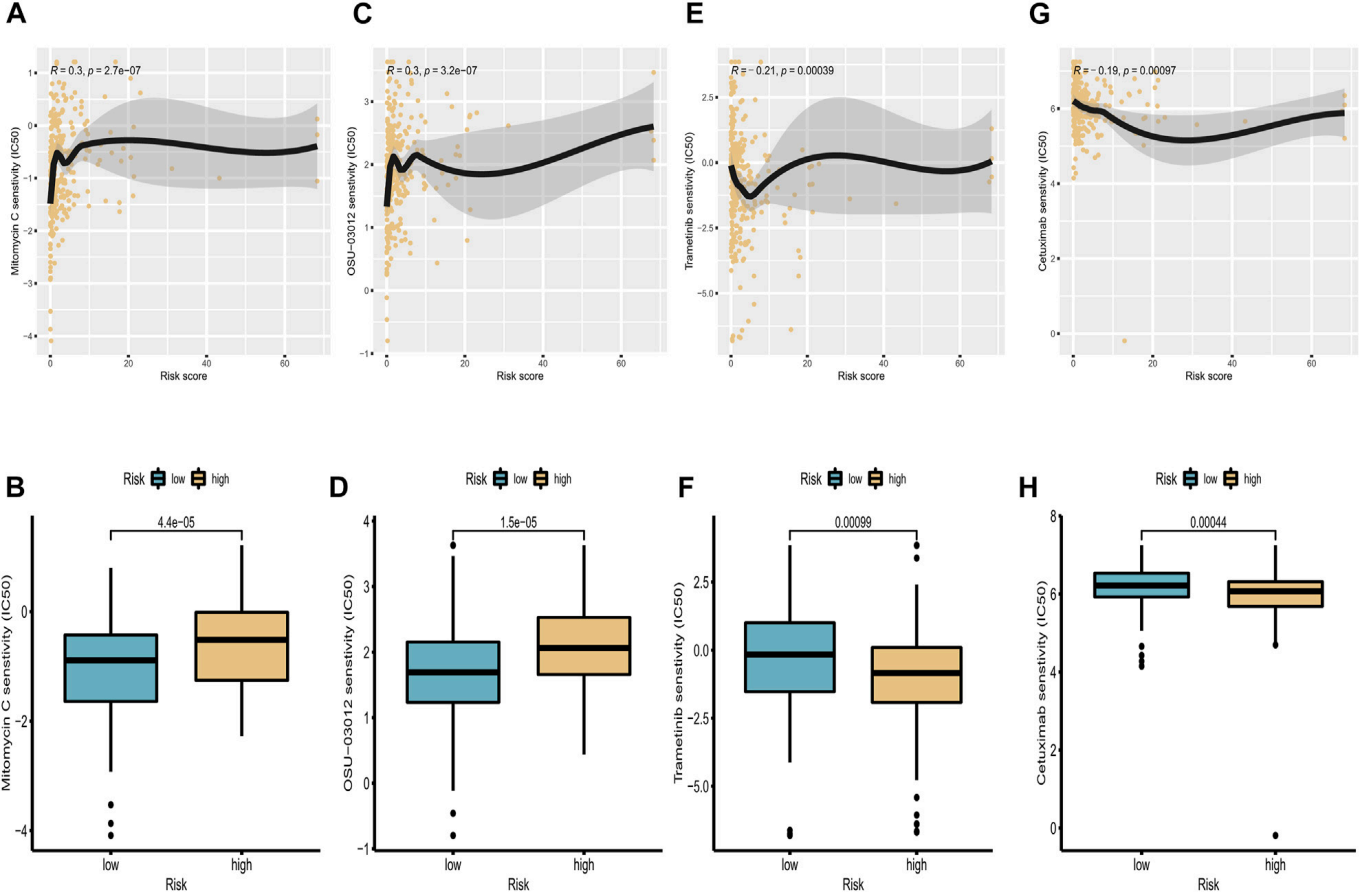
Correlation analysis of targeted drugs. **(A,C,E,G)** Correlations between IC50 levels of four targeted drugs (osu-03012, mitomycin C, trametinib, and cetuximab) and expression of cuproptosis-related lncRNAs. **(B,D,F,H)** Differences in sensitivity to osu-03012, mitomycin C, trametinib, and cetuximab between high-risk and low-risk groups.

Pharmacotherapy played a vital role in the treatment of patients with CC. To investigate the clinical utility of our signature for guiding decision-making in the treatment of CC, we investigated the association between the risk score and the IC50 values of four commonly used therapeutic drugs. The estimated IC50 levels of these four drugs in the two groups were compared and visualized using scatterplots and boxplots. We found that osu-03012 and mitomycin c may be more suitable for patients in the low-risk group (Figures 9A–D), whereas trametinib and cetuximab may be candidate drugs for the high-risk group (Figures 9E–H). These results indicate that our proposed risk signature may be useful as an indicator of drug sensitivity.

### Validation of the cuproptosis-related prognostic signature

**FIGURE 10:**
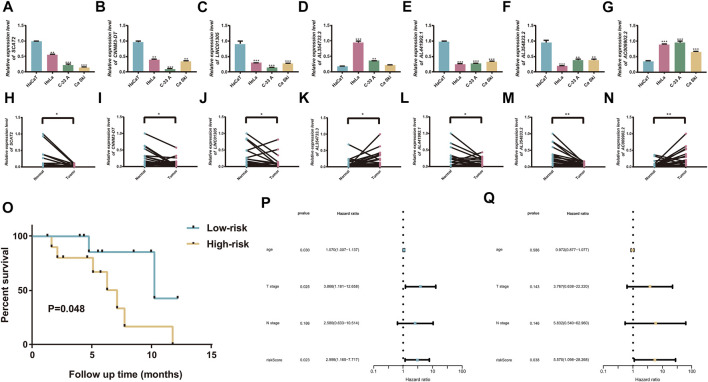
Validation of the cuproptosis-related prognostic signature. **(A–G)** Comparison of the expression of seven candidate lncRNAs between cervical tumor and normal cells. **(H–N)** twenty cercival cancer and matched adjacent normal tissues. **(O)** K-M curves exhibited that the RS was significantly associated with the overall survival. **(P)** Univariate Cox regression analysis demonstrated that the signature and T stage were significantly associated with prognosis. **(Q)** Multivariate Cox regression analysis revealed that RS could function as an independent factor in CC. *p < 0.05, **p < 0.01, and ***p < 0.001.

The expression levels of the seven cuproptosis-related lncRNAs were determined using qRT-PCR in normal cervical cells and CC cells. The results showed that AC009902.2 and AL354733.3 were significantly upregulated in tumor cells compared with normal tissues, whereas AL441992.1, LINC01305, AL354833.2, CNNM3-DT, and SCAT2 were downregulated in CC cells (Figures 10A–G). In sample pairs retracted from CC patients in our hospital, we additionally verified the expression levels of these 7 lncRNAs. Clinical samples exhibited similar expression trends. (Figures 10H–N). Subsequently, twenty CC patients were divided into high/low risk groups according to the median RS calculated based on the signature. Kaplan-Meier analysis revealed that the OS of the high-risk group was significantly lower than that of the low-risk group.

The expression levels of the seven cuproptosis-related lncRNAs were detected by RT-qPCR in 20 cervical cancer patients with tumor tissues and paracological tissues. Kaplan-Meier analysis revealed that the OS of the high-risk group was significantly lower than that of the low-risk group (*p* = 0.048; Figure 10O). The Cox regression analyses showed that the RS could serve as an independent prognostic predictor for CC patients, confirming the reliability of the signature (Figures 10P–Q).

## Discussion

CC is among the most common malignancies in women and remains the leading cause of cancer-related deaths in women in most developing countries (Sung et al., 2021). The current standard treatment for CC includes radical hysterectomy and lymph node dissection and/or radiotherapy for early-stage patients (Landoni et al., 1997; Gadducci et al., 2003), and external radiotherapy, concurrent cisplatin chemotherapy, and brachytherapy for patients with advanced disease (Morris et al., 1999; Sturdza et al., 2016). Despite advances in treatment, owing to the heterogeneity of the tumors, the prognosis of CC patients varies significantly even among those with similar clinical features undergoing similar treatment regimens, suggesting that existing classifications and clinicopathologic features are insufficient to predict patient prognosis. Therefore, the search for new classifier models to stratify patients with varied risk profiles and predict therapeutic drug response is crucial.

Copper is a mineral nutrient closely associated with cellular functions including mitochondrial respiration, antioxidant defense, and biosynthetic processes (Que et al., 2008). Numerous studies have demonstrated that copper is also associated with cancer cell growth, proliferation, and metastasis (Pan et al., 2002; Ding et al., 2015; Blockhuys et al., 2017; Stepien et al., 2017; Shanbhag et al., 2019). However, it is crucial that cellular copper is present in moderate amounts, as excess copper mediates cytotoxicity, leading to cell death (Kahlson and Dixon, 2022). Tsvetkov and colleagues proposed that cuproptosis, which differs from other forms of regulated cell death caused by oxidative stress, is triggered by mitochondrial protein aggregation accompanied by intracellular copper accumulation. Copper ion carriers such as disulfiram and everolimus can be used to effectively treat various human cancers by inducing cuproptosis, suggesting that cuproptosis may be critical in the development and occurrence of cancer. LncRNAs have been shown to play an essential part in cancer by regulating cellular signaling cascade responses (Sanchez Calle et al., 2018). The predictive power of lncRNA-based signatures reported in recent studies suggests that lncRNAs are good indicators of CC prognosis (Mao et al., 2019; Wu et al., 2019; He et al., 2020). However, there is still a lack of cuproptosis-related lncRNAs for predicting the prognosis of CC patients.

This study included a total of 285 CC patients from TCGA, who were divided into a training set and the internal validation set. By co-expression correlation analysis, we identified 460 lncRNAs correlated with cuproptosis, of which seven lncRNAs (AL441992.1, LINC01305, AL354733.3, AL354833.2 AC009902.2, CNNM3-DT, and SCAT2) were screened by LASSO regression analysis to construct a prognostic signature. LINC01305 has been shown to regulate the target gene HTR3A mRNA through interaction with IGF2BP2 and IGF2BP3, thereby participating in the metastasis and proliferation of esophageal squamous cell carcinoma (Huang et al., 2021a). LINC01305 can mediate the RNA-binding protein KHSRP and activate the Wnt signaling pathway through upregulation of β-linked protein, TCF7, and CCND2, thereby participating in CC cell stemness (Huang et al., 2021b). Chen et al. constructed a cervical cancer prognosis model based on immune-associated lncRNAs, wherein AL441992.1 was a protective factor in cervical cancer, which was consistent with our research (Chen et al., 2020a). Some researchers have selected AL354733.3 into their prognostic models as a protective gene to predict survival in oral and oropharyngeal squamous cell carcinoma (Jiang et al., 2021). However, in our risk signature, AL354733.3 is a risk gene, and the relative expression level is higher in cancer cells and tissues, implying that the function of AL354733.3 in cancer merits further exploration. Up to now, the function and in-depth molecular interactions of the remaining four lncRNAs have not been reported.

The OS and PFS survival curves demonstrated the power of the signature in predicting prognosis of patients with CC. Univariate and multivariate Cox analyses showed that risk score were independent prognostic factors. ROC curves demonstrated the superior specificity and sensitivity of the signature in predicting patients’ 1, 3, and 5-year survival probability in comparison with AJCC stage. Nomograms were also constructed based on risk score and prognosis-related clinical characteristics to predict the probability of OS in patients with CC. The calibration plots showed excellent agreement between the nomograms’ anticipated survival probability and the actual survival outcome of patients, confirming the predictive accuracy of the nomograms. In addition, the results of PCA showed that patients with CC could be distinguished clearly based the risk score from the prognostic signatures. Recently, researchers have examined the relationship between lncRNAs and immune or cell death-related phenotypes in CC and established prognostic diagnostic models. As a result, the immune-related gene diagnostic model’s 1-year AUC was 0.780, 0.695, 0.793, and 0.77 respectively (Mao et al., 2019; Chen et al., 2020a; Zheng et al., 2020; Zhong et al., 2021). The 1-year AUC value for the autophagy-associated lncRNA diagnostic signature was 0.710 (Jiang et al., 2021). In this study, the average AUC values of 1-, 3- and 5-year prognosis predictions reached 0.807, 0.824 and 0.793. Although the study of Chen et al. showed that the 1-year AUC value of the immune-related lncRNA diagnostic signature was 0.884, its 3-year and 5-year AUC values were 0.778 and 0.781, which were smaller than the signature in this study (Chen et al., 2020b). Generally, our prognostic signature performs better than these known prognostic biomarkers.

GO and KEGG analysis were conducted to elucidate the biological activities and pathways related to the signature. The results showed that the DEGs were significantly enriched in pathways including cytokine–cytokine receptor interaction, chemical carcinogenesis-reactive oxygen species, and oxidative phosphorylation. Reactive oxygen species (ROS) are peroxides that drive cellular regulatory pathways by altering cell-signaling proteins and can affect the tumor microenvironment (Zhang et al., 2016). Studies have shown that abnormally elevated ROS levels in cancer cells persistently lead to downregulation of the cellular antioxidant enzyme system, promoting tumor proliferation, survival, and metastasis (Aggarwal et al., 2019; Babu and Tay, 2019; Wang et al., 2021). Related studies have shown that the metabolism of cancer cells is closely related to the oxidative phosphorylation pathway in mitochondrial respiration, and reversing the Warburg effect also supports the function of oxidative phosphorylation in cancer (LeBleu et al., 2014; Tango et al., 2020). Li et al. (2021) found that lncRNA OIP5-AS1 was upregulated in CC tissues and promoted the Warburg effect through induction of the miR-124-5p/IDH2/HIF-1α pathway, leading to proliferation of CC cells. By contrast, studies in RIP1-Tag2 mice showed that intracellular copper expression levels regulate cancer cell proliferation and oxidative phosphorylation through the formation of cytochrome oxidase catalysis (Ishida et al., 2013).

Gene co-expression network analysis has been widely used to assess the potential roles of lncRNAs. For example, Shi et al. constructed a ceRNA network of lncRNAs, miRNAs, and mRNA to confirm its involvement in HCC prognosis through “protein kinase activity,” “cell morphogenesis involved in differentiation,” and other pathways (Shi et al., 2021). Li et al. also performed co-expression analysis to explore the regulatory relationship between lncRNAs and mRNAs in CC (Jiang et al., 2019). In this study, we constructed a triple-ceRNA regulatory network based on cuproptosis-related lncRNAs. Subsequently, enrichment analysis was carried out to explore the functions of target mRNAs, and we found that these target genes were significantly enriched in several immune-related pathways, including immunoregulatory interactions between a lymphoid and a non-lymphoid cell and leukocyte cell–cell adhesion, indicating that cuproptosis-related lncRNAs may have an important role in the tumor microenvironment of CC.

The ssGSEA results indicated that patients in the high-risk group had lower levels of protective immune cell infiltration, including infiltration of CD8 T cells and T helper 1 (Th1) cells, indicating a poor prognosis for patients in this group. Previous studies have shown that solid infiltration of memory CD8 T cells, NK cells, and Th1 cells is associated with good prognosis (Fridman et al., 2012; Barnes and Amir, 2018). Moreover, various immune functions were activated in the low-risk group, including HLA, checkpoints, and inflammation-promotion. Studies have shown that HLA plays an important part in the presentation of neoantigens. The infiltration of CD8^+^ T and NK cells and the expression of immune checkpoints are almost uniformly positively correlated with the expression of HLA genes in all cancer types (Schaafsma et al., 2021), which is in accordance with our findings above. In the past few years, immune checkpoint inhibitors (ICIs) have emerged as a new approach with significant efficacy in the treatment of CC. Combination therapy with ICIs can overcome tumor resistance and improve treatment outcomes (Patel and Minn, 2018; Zhang et al., 2022). It has now been shown that inhibitors of PD-1 and CTLA4 can harness the host immune system to achieve antitumor effects in the treatment of CC (Jiménez-Lima et al., 2020; Wendel Naumann and Leath, 2020). Therefore, identifying patients who are candidates for ICI therapy in clinical practice is critical. We conducted differential expression analysis of four immune checkpoints, PD-1, CTLA4, LAG-3, and TIGIT, between the high- and low-risk groups. The results showed that all four immune checkpoint molecules were overexpressed in the low-risk group, which demonstrated that ICI treatment is more suitable for patients with low-risk scores.

In addition, we conducted drug sensitivity analysis of four agents between the two risk groups, including mitomycin c, osu-03012, trametinib and cetuximab. Trametinib is a reversible, highly selective allosteric inhibitor of MEK1/MEK2 activation. Relevant research showed, trametinib combined with PI3K/mTOR dual inhibitor dactylitis can eliminate the enhancement of nuclear receptor related-1 protein (Nurr1) to cervical cancer cell aggressiveness by upregulating p21 and p27 expression and inhibiting MMP9 and KLF4 expression (Wan et al., 2021). Cetuximab is a kind of epidermal growth factor receptor (EGFR) inhibitor (Akimoto et al., 1999; Bianco et al., 2000; Huang and Harari, 2000; Milas et al., 2000). A phase II study in patients with squamous cell or non-squamous cervical cancer found that cetuximab was more effective in patients with squamous cell histotypes (Farley et al., 2011). Yun Hee Kang et al. found that mitomycin c can inhibit cervical cancer cell growth by downregulating repair genes such as Brca1 and its binding proteins or associated proteins such as Ku70 binding protein (KUB3) and Brca1-associated protein (Brca1AP) (Kang et al., 2010). OSU-03012 is a novel small molecule inhibitor of the PI3K/Akt signaling pathway that inhibits tumor growth in a variety of ways (Ding et al., 2021). While the role of OSU-03012 in CC with high PI3K/Akt activation has not been studied, our findings may provide new therapeutic ideas for its treatment.

The study had some limitations. First, data used for construction of the risk signature were obtained only from TCGA; future prospective studies with larger clinical cohorts will be needed in the future to cross-validate the signature’s prognosis reliability and accuracy. Second, the mechanism underlying the relationship between cuproptosis-related lncRNAs and CC prognosis remains unclear and needs to be elucidated through further research.

## Conclusion

In conclusion, we constructed a prognostic signature of seven cuproptosis-related lncRNAs by systematically studying the expression profiles of cuproptosis-related lncRNAs and clinical information of patients with CC. The predictive performance of the prognostic signature was evaluated and validated in the internal validation set and the entire dataset. In addition, our risk signature could predict response to targeted therapy and immunotherapy, which is critical in making individualized decisions in clinical treatment.

## Data Availability

The original contributions presented in the study are included in the article/Supplementary Material, further inquiries can be directed to the corresponding author.
