# Supplementary figures and images for: Signature of seven cuproptosis-related lncRNAs as a novel biomarker to predict prognosis and therapeutic response in cervical cancer
Source: Front Genet. 2022 Sep 20;13:989646. doi: 10.3389/fgene.2022.989646 (PMC9530991; doi:10.3389/fgene.2022.989646)

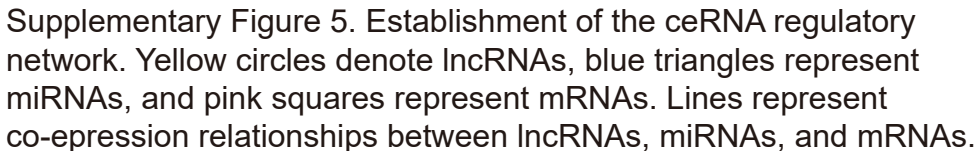

Supplement: Supplementary file 2 [file Image5.PDF]
