# Supplementary material for: Signature of seven cuproptosis-related lncRNAs as a novel biomarker to predict prognosis and therapeutic response in cervical cancer
Source: Front Genet. 2022 Sep 20;13:989646. doi: 10.3389/fgene.2022.989646 (PMC9530991; doi:10.3389/fgene.2022.989646)
Supplement: Supplementary file 3 [file Table1.DOCX]

| cuprotosis-related gene |
| --- |
| NFE2L2 |
| NLRP3 |
| ATP7B |
| ATP7A |
| SLC31A1 |
| FDX1 |
| LIAS |
| LIPT1 |
| LIPT2 |
| DLD |
| DLAT |
| PDHA1 |
| PDHB |
| MTF1 |
| GLS |
| CDKN2A |
| DBT |
| GCSH |
| DLST |
