# Supplementary material for: Signature of seven cuproptosis-related lncRNAs as a novel biomarker to predict prognosis and therapeutic response in cervical cancer
Source: Front Genet. 2022 Sep 20;13:989646. doi: 10.3389/fgene.2022.989646 (PMC9530991; doi:10.3389/fgene.2022.989646)
Supplement: Supplementary file 4 [file Table8.DOCX]

**Table 1 Characteristics of training set, internal validation set, and entire set**

| **Characteristics** | **Training Set**  **N=143** | **Internal validation set**  **N=142** | **Entire Set**  **N=285** | **P Value** |
| --- | --- | --- | --- | --- |
|  | **Number(%)** | **Number(%)** | **Number(%)** |  |
| **Age** |  |  |  | **0.2576** |
| **≤65** | **130(90.9)** | **122(85.9)** | **252(88.4)** |  |
| **>65** | **13(0.1)** | **20(14.1)** | **33(11.6)** |  |
| **Grade** |  |  |  | **0.8908** |
| **1-2** | **76(53.1)** | **69(48.6)** | **145(50.9)** |  |
| **3** | **58(40.6)** | **54(38.0)** | **112(39.3)** |  |
| **Unknown** | **9(6.3)** | **19(13.4)** | **28(9.8)** |  |
| **Stage** |  |  |  | **0.2610** |
| **I-II** | **49(33.3)** | **48(33.8)** | **97(34.0)** |  |
| **III-IV** | **1(0.7)** | **3(2.1)** | **4(1.4)** |  |
| **Unknown** | **93(65.0)** | **91(64.1)** | **184(64.6)** |  |
| **Tumor size** |  |  |  | **0.6561** |
| **T1+T2** | **104(72.7)** | **96(67.6)** | **200(70.2)** |  |
| **T3+T4** | **11(7.7)** | **15(10.6)** | **26(9.1)** |  |
| **Unknown** | **28(19.6)** | **31(21.8)** | **59(20.7)** |  |
| **Lymph node metastasis** |  |  |  | **0.6981** |
| **N0** | **65(45.4)** | **60(42.3)** | **125(43.9)** |  |
| **N1** | **26(18.2)** | **28(19.7)** | **54(18.9)** |  |
| **Unknown** | **52(36.4)** | **54(38.0)** | **106(37.2)** |  |
| **Distant metastasis** |  |  |  | **0.7564** |
| **M0** | **55(38.5)** | **51(35.9)** | **106(37.2)** |  |
| **M1** | **4(2.8)** | **6(4.2)** | **10(3.5)** |  |
| **Unknown** | **84(58.7)** | **85(59.9)** | **169(59.3)** |  |
| **Death** |  |  |  | **1** |
| **No** | **107(74.8)** | **107(75.4)** | **214(75.1)** |  |
| **Yes** | **36(25.2)** | **35(24.6)** | **71(24.9)** |  |

***Statistical analysis in age, grade, stage, tumor size, lymph node metastasis, distant metastasis, and Death between training set and internal validation set**
