# Supplementary material for: Signature of seven cuproptosis-related lncRNAs as a novel biomarker to predict prognosis and therapeutic response in cervical cancer
Source: Front Genet. 2022 Sep 20;13:989646. doi: 10.3389/fgene.2022.989646 (PMC9530991; doi:10.3389/fgene.2022.989646)
Supplement: Supplementary file 5 [file Image4.PDF]

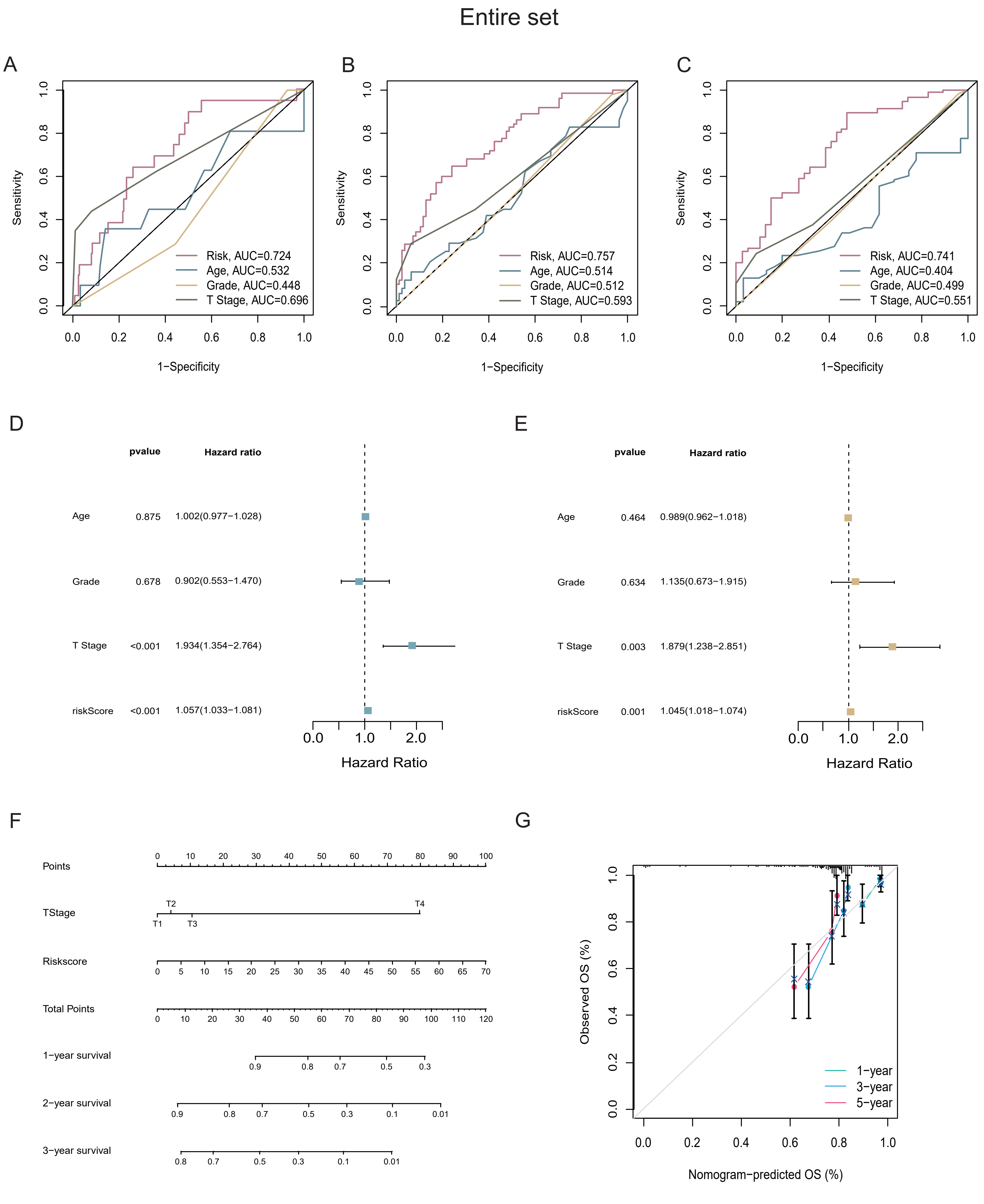

Figure S4. Construction and validation of the prognostic nomogram in the entire set. (A-C) AUC values of nomogram points at 1-year, 3-years, and 5-years were compared with other clinical parameters, including age, grade, stage, and T stage. (D, E) Forest plots summarizing the results of univariate and multivariate Cox analyses of risk scores and clinicopathological features. (F) The nomogram was plotted based on the signature and T Stage. (G) Calibration plot for internal validation of the nomogram. Survival is depicted on the Y-axis, and nomogram-predicted survival is shown on the X-axis. Besides the dashed diagonal line, green, blue, and red lines represent the 1-year, 3-year, and 5 - year observed nomograms.
