# Supplementary material for: Signature of seven cuproptosis-related lncRNAs as a novel biomarker to predict prognosis and therapeutic response in cervical cancer
Source: Front Genet. 2022 Sep 20;13:989646. doi: 10.3389/fgene.2022.989646 (PMC9530991; doi:10.3389/fgene.2022.989646)
Supplement: Supplementary file 9 [file Table3.DOCX]

| **lncRNA_symbol** | **HR** | **95% CI lower** | **95% CI upper** | **Coef** | **p value** |
| --- | --- | --- | --- | --- | --- |
| **AL441992.1** | 0.5240 | 0.3152 | 0.8710 | -0.7587 | 0.0127 |
| **LINC01305** | 0.6278 | 0.4423 | 0.8910 | -0.8764 | 0.0092 |
| **AL354733.3** | 4.2003 | 1.5345 | 11.4972 | 2.5069 | 0.0052 |
| **AL354833.2** | 0.1049 | 0.0151 | 0.7286 | -2.9154 | 0.0226 |
| **AC009902.2** | 1.7695 | 1.0136 | 3.0892 | 0.6460 | 0.0447 |
| **CNNM3-DT** | 0.3044 | 0.1260 | 0.7353 | -0.8982 | 0.0082 |
| **SCAT2** | 0.5482 | 0.3010 | 0.9697 | -0.6404 | 0.0389 |

**Table S3: The signature of 7 cuproptosis-related lncRNAs with prognostic value in CC**
