# Supplementary material for: Signature of seven cuproptosis-related lncRNAs as a novel biomarker to predict prognosis and therapeutic response in cervical cancer
Source: Front Genet. 2022 Sep 20;13:989646. doi: 10.3389/fgene.2022.989646 (PMC9530991; doi:10.3389/fgene.2022.989646)
Supplement: Supplementary file 10 [file Table4.DOCX]

| **Category** | **ID** | **Term** | **Count** | **P value** |
| --- | --- | --- | --- | --- |
| BP | GO:0002443 | leukocyte mediated immunity | 16 | 8.58E-08 |
| BP | GO:0002449 | lymphocyte mediated immunity | 14 | 1.84E-07 |
| BP | GO:0002455 | humoral immune response mediated by circulating immunoglobulin | 9 | 1.90E-07 |
| BP | GO:0006956 | complement activation | 10 | 2.80E-08 |
| BP | GO:0006958 | complement activation, classical pathway | 9 | 7.10E-08 |
| BP | GO:0006959 | humoral immune response | 15 | 7.01E-09 |
| CC | GO:0009897 | external side of plasma membrane | 20 | 8.64E-12 |
| CC | GO:0019814 | immunoglobulin complex | 17 | 1.57E-15 |
| CC | GO:0042101 | T cell receptor complex | 10 | 6.94E-08 |
| CC | GO:0042571 | immunoglobulin complex, circulating | 9 | 2.59E-09 |
| CC | GO:0062023 | collagen-containing extracellular matrix | 10 | 0.000664844 |
| CC | GO:0098802 | plasma membrane signaling receptor complex | 10 | 4.71E-05 |
| MF | GO:0003823 | antigen binding | 12 | 1.69E-09 |
| MF | GO:0004030 | aldehyde dehydrogenase [NAD(P)+] activity | 3 | 0.00015397 |
| MF | GO:0005539 | glycosaminoglycan binding | 8 | 0.000160189 |
| MF | GO:0030546 | signaling receptor activator activity | 13 | 2.79E-05 |
| MF | GO:0034987 | immunoglobulin receptor binding | 9 | 2.80E-09 |
| MF | GO:0048018 | receptor ligand activity | 13 | 2.36E-05 |
