# Supplementary material for: Signature of seven cuproptosis-related lncRNAs as a novel biomarker to predict prognosis and therapeutic response in cervical cancer
Source: Front Genet. 2022 Sep 20;13:989646. doi: 10.3389/fgene.2022.989646 (PMC9530991; doi:10.3389/fgene.2022.989646)
Supplement: Supplementary file 13 [file Image1.PDF]

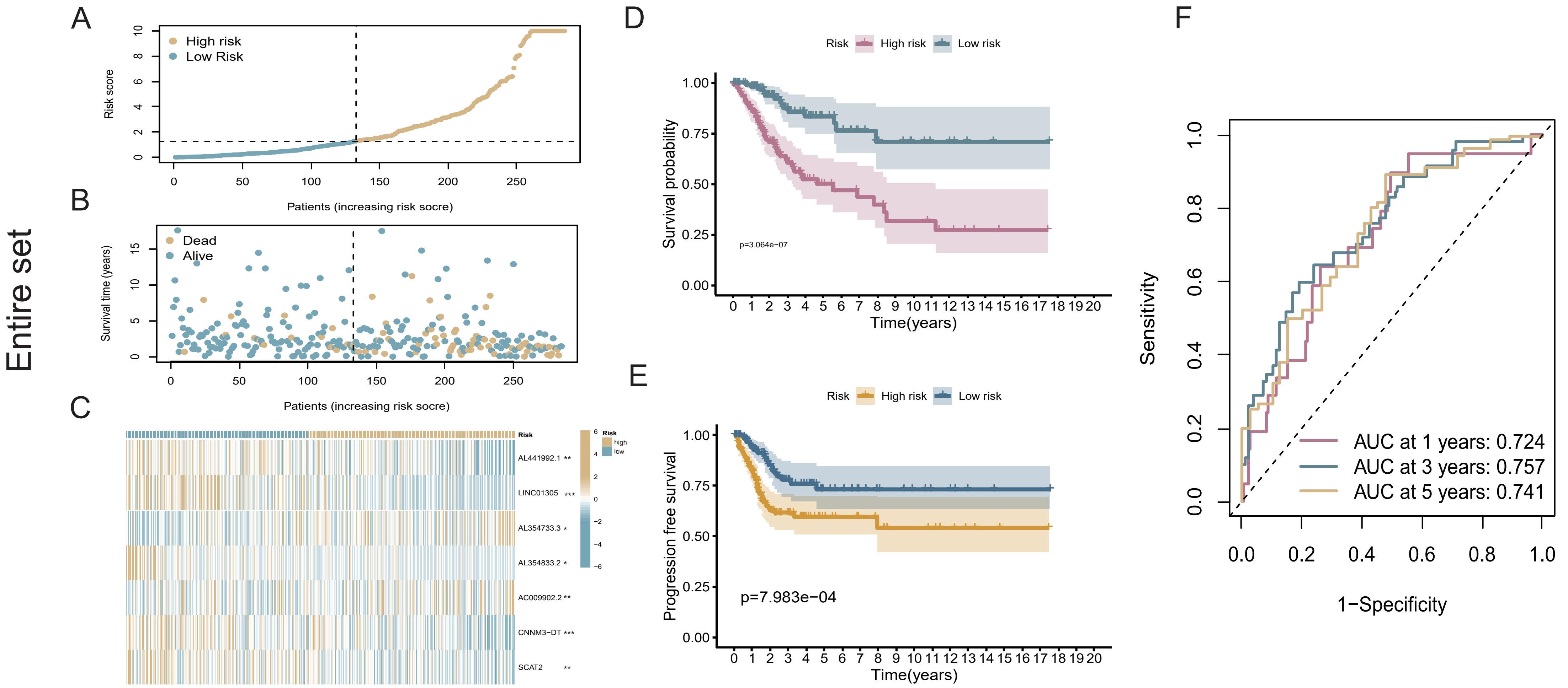

Figure S1. Validation of the prognostic ability of the signature in the entire set. (A) Risk curves based on the risk scores in high-risk and low-risk groups. (B) Scatter plot showing each patient's survival status. Blue represents survival, yellow represents death. (C) Heatmap showing the expression levels of cuproptosis-related lncRNAs in high-risk and low-risk groups. (D, E) Kaplan–Meier survival analysis showing the difference in patient prognosis between the high-risk and low-risk groups with respect to OS (D) and PFS (E). (F) Time-dependent ROC curves for 1 - , 3 - , and 5 - year OS. *p<0.5; **p<0.01; ***p<0.001
